# Identification of single amino acid differences in uniformly charged homopolymeric peptides with aerolysin nanopore

**DOI:** 10.1038/s41467-018-03418-2

**Published:** 2018-03-06

**Authors:** Fabien Piguet, Hadjer Ouldali, Manuela Pastoriza-Gallego, Philippe Manivet, Juan Pelta, Abdelghani Oukhaled

**Affiliations:** 1 LAMBE UMR 8587, Université de Cergy-Pontoise, 95300 Pontoise, France; 2 APHP, Centre de Ressources Biologiques BB-0033-00064, Plateforme de Bio-Pathologie et de Technologies Innovantes en santé, Hôpital Lariboisière, 75010 Paris, France; 3 INSERM UMR-S942, Hôpital Lariboisière, 75010 Paris, France; 4 LAMBE UMR 8587, Université d’Evry-Val-d’Essonne, 91000 Evry, France

## Abstract

There are still unmet needs in finding new technologies for biomedical diagnostic and industrial applications. A technology allowing the analysis of size and sequence of short peptide molecules of only few molecular copies is still challenging. The fast, low-cost and label-free single-molecule nanopore technology could be an alternative for addressing these critical issues. Here, we demonstrate that the wild-type aerolysin nanopore enables the size-discrimination of several short uniformly charged homopeptides, mixed in solution, with a single amino acid resolution. Our system is very sensitive, allowing detecting and characterizing a few dozens of peptide impurities in a high purity commercial peptide sample, while conventional analysis techniques fail to do so.

## Abstract (web summary)

Existing methods used for peptide analysis suffer from low sensitivity and specificity. Here the authors demonstrate the nanopore-based size-discrimination and purity analysis of short homopolymeric peptides with a single amino acid resolution.

## Introduction

Recently, the concept of personalized medicine has evolved towards the analysis of rare cells (tumor and endothelial cells, cancer stem cells, etc.) and extracellular vesicles circulating in the blood, containing a few copies of biomarkers. Among the biomarkers there are short signal peptides that are able to modulate the cancer cells aggressiveness more or less upon their ability to be processed by proteolysis driven by N-end rules pathways^1^. Classical metabolomics and proteomics techniques, such as mass spectrometry (MS), High Performance Liquid Chromatography (HPLC), or Nuclear Magnetic Resonance (NMR), suffer a lack of reproducibility and sensitivity that prohibit their use for medical diagnostic^2,3^. In addition, these techniques are too expensive, time consuming and heavy to handle in the view of developing a portable medical device application^3^. In the industry context, pharmaceutical peptides manufacturing requires a high purity level and is still waiting for high-sensitivity analytical methods addressing Good Manufacturing Practice issues such as impurities profile characterization^4^. Nanopore single-molecule technology^5^ seems to be an excellent alternative for addressing the above issues. For more than two decades, the nanopore technology has been essentially applied to DNA^6^. The development of nanopore-based DNA sequencing is now achieved and commercialized. The current challenge is the application of nanopore technology to peptides and proteins identification, fingerprinting and sequencing.

To date, nanopores were successfully used to detect protein or peptides either through biological nanopores^7–39^ or through artificial nanopores^40–59^. Protein or peptides were probed either inside a natural nanopore^21,29,35^ or outside a natural nanopore with an aptamer^21,22^ or with a ligand^7^. Pioneering studies were devoted to the dynamics of peptides transport^9,10,13,14,16,30^, and then to the dynamics of unfolded proteins transport^18,31,48^ through the nanopore. Others studies focused on protein-antibodies complex^32,41,42^, on intrinsically disordered proteins^49^, on enzymatic reactions^15,17,23,60,61^ and on the aggregation of amyloidogenic peptides^36,47,55^. The unfolding of proteins was investigated, either in the presence of an unfolding agent^12,31,44^, or by varying temperature^24^, or with an electrical force^50,62^, or with a molecular motor such as an unfoldase^27^. Nanopores were also used to study the structure of peptides^8^, conformational changes of proteins^12,39,44,54,57^, post-translational modification of proteins^52,58^ and to estimate the shape, volume, charge, rotational diffusion coefficient and dipole moment of individual proteins^63^.

In the context of nanopore-based protein sizing, glass nanopores were used to determine folded protein sizes ranging from 12 to 480 kDa^51,53^. The best discrimination resolution obtained with this approach was a difference of 4 kDa in the case of the shortest proteins, and a difference of 50 kDa in the case of the longest proteins^51,53^. Then solid-state nanopores were demonstrated to enable the detection of proteins as small as 8.5 kDa^54^ and the discrimination between two different proteins with a resolution of 1.7 kDa^57^. Regarding biological nanopores, Fragaceatoxin C (FraC) channels were recently successfully used to discriminate peptides and proteins as a model biomarkers ranging from 25 kDa down to 1.3 kDa^38^. In the same context, *α*-hemolysin nanopore equiped with Au_25_(SG)_18_ clusters was used to increase the on-rate and off-rate of peptides to the pore in order to improve the mass resolution of the nanopore sensor^37^. Thus, while biological nanopores were previously used for size-discriminating DNA hairpins^64^, synthetic polymers^65–67^ and oligonucleotides^68^ with monomeric resolution, the single-amino-acid size-discrimination between several (more than two) peptides mixed in solution that differ by a single amino acid and the characterization of rare peptide impurities were not demonstrated, regardless of the pore used^9,10,20,30,37,38,65,66,68^. In previous works using biological nanopores, there was no more than two peptides differing by one amino acid among the different peptides used^10,37,38^.

Here, we demonstrate the proof-of-concept of size-discrimination of short uniformly charged homopeptides, mixed in solution or independently, with a single amino acid resolution by using the recombinant aerolysin nanopore without any physical or chemical modifications. The sensitivity of this pore allows detecting, with a single amino acid resolution, peptide fragments of different lengths present in a high purity commercial peptide sample. We also show the discrimination between two homopolymeric- and one heteropolymeric peptides having the same length. Our system also enables the real-time monitoring of the evolution of the peptide populations with a single amino acid resolution during the enzymatic degradation of a peptide sample. These findings open new perspectives, in particular towards the use of nanopore technology for the size-discrimination and sequence recognition of peptides and proteins.

## Results

### Single amino acid resolution size-discrimination of peptides

**Fig. 1:**
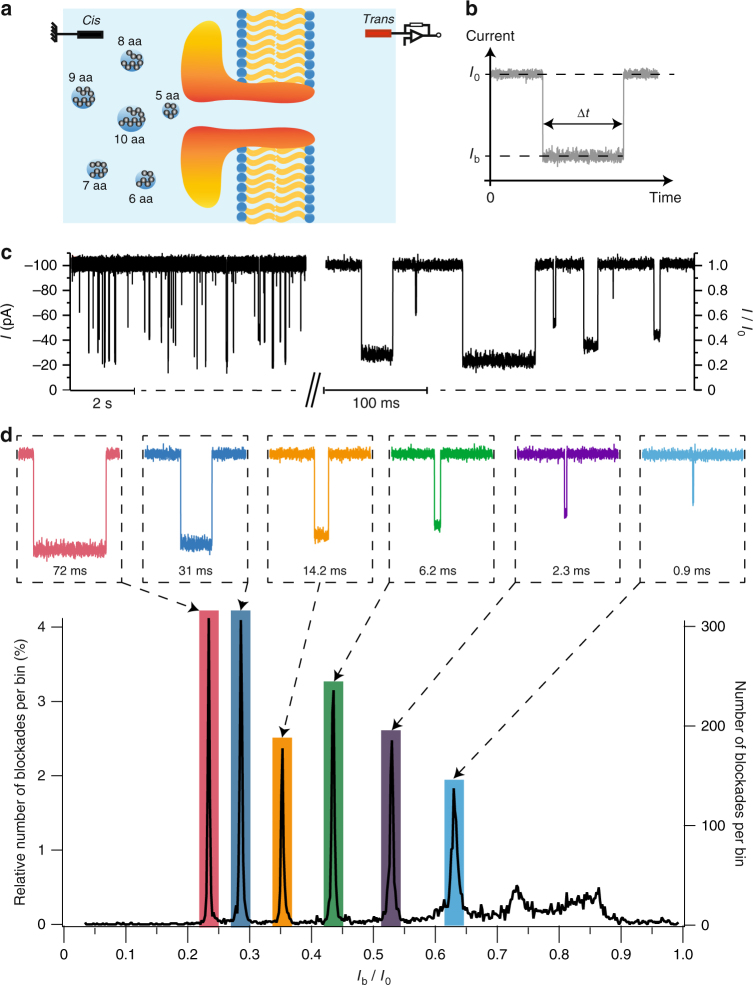
Detection of a mixture of arginine peptides of different lengths. **a** Schematics of the experimental setup (not to scale). A single wild-type aerolysin nanopore was inserted in a lipid bilayer separating the *cis*- and *trans*-compartments filled with an electrolyte solution. An external voltage was applied on the *trans*-side of the bilayer, the *cis*-side being at voltage ground. A mixture of arginine peptides of different lengths (5, 6, 7, 8, 9, and 10 amino acids) was added on the *cis*-side of the bilayer. **b** Illustration of a typical current blockade event. The interaction of a peptide with the nanopore is detected as a partial and temporary blockade of the electrical current flowing through the nanopore from its open-pore current value *I*_0_ to the blockade current value *I*_b_. The current blockade duration Δ*t* corresponds to the duration of the peptide/nanopore interaction. **c** Portion of a typical current versus time recording through aerolysin nanopore in presence of an equimolar mixture (1 μM) of arginine peptides of different lengths (5, 6, 7, 8, 9, and 10 amino acids) in KCl 4 M HEPES 5 mM pH = 7.5 at −50 mV and at 20 °C. The left axis indicates the current *I* values (negative current values under a negative applied voltage) and the right axis indicates the relative current *I*/*I*_0_ values. An enlargement of the current trace shows typical current blockades of different amplitudes and durations. **d** Histogram of the relative blockade current *I*_b_/*I*_0_ values corresponding to the current trace in (**c**). The right axis indicates the number of blockades per bin and the left axis indicates the relative number of blockades per bin (i.e., the ratio of the number of blockades per bin on the total number of blockades). The histogram reveals the existence of at least 6 distinct populations corresponding to preferred relative blockade current values. Inserts on the top show a typical current blockade event and indicate the mean blockade duration for each population

A schematics of the experimental setup is shown in Fig. 1a. A single wild-type aerolysin nanopore was inserted in a lipid bilayer separating the *cis*-compartments and *trans*-compartments filled with an electrolyte solution. An external voltage was applied on the *trans*-side of the bilayer, the *cis*-side being at voltage ground. In absence of analytes, a stable ionic current of mean value *I*_0_ flowing through the nanopore was measured. The addition of an equimolar mixture of arginine peptides of 6 different lengths differing by a single amino acid in length (5, 6, 7, 8, 9, 10 amino acids) on the *cis*-side of the bilayer induced well-resolved current blockades of different amplitudes and durations under a negative applied voltage (Fig. 1a,c), in agreement with the positive electrical charge of arginine peptides under our experimental conditions. In contrast, no current blockades were observed either when the peptides were added on the *cis*-side under a positive applied voltage, or when the peptides were added on the *trans*-side regardless of the voltage polarity (Supplementary Fig. 1).

The histogram of the relative blockade current *I*_b_/*I*_0_ values induced by the mixture of arginine peptides of 6 different lengths is shown in Fig. 1d. Well-separated peaks corresponding to distinct populations with preferred *I*_b_/*I*_0_ values are observed. For each of the 6 main populations, the mean relative blockade current is (mean value of a gaussian fit of the peak +/− one standard deviation): 0.234 +/− 0.001, 0.286 +/− 0.002, 0.353 +/− 0.002, 0.435 +/− 0.002, 0.530 +/− 0.002, 0.631 +/− 0.004. The interval between two consecutive peaks is at least 25-fold greater than the peaks widths. The mean blockade duration of each population increases by almost two orders of magnitude as the relative blockade current value decreases, from 0.9 ms for the shallowest population to 72 ms for the deepest population.

**Fig. 2:**
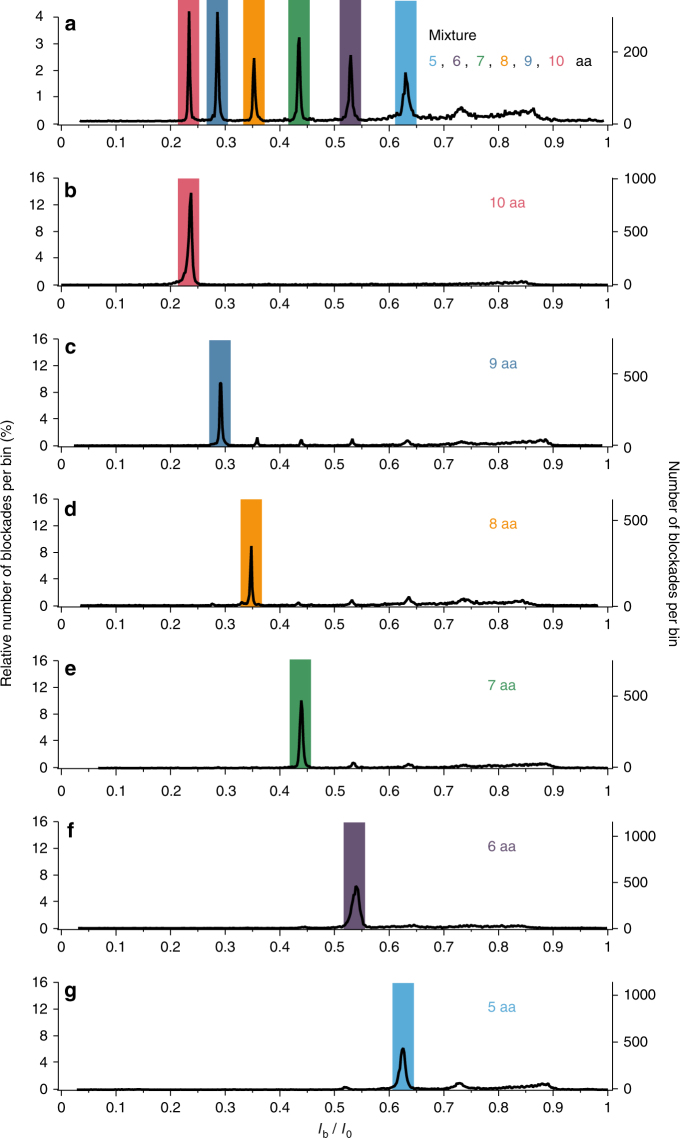
Size-discrimination of arginine peptides with a single amino acid resolution. Histograms of the relative blockade current *I*_b_/*I*_0_ values in the case of the interaction of aerolysin nanopore with: **a** an equimolar mixture of arginine peptides of different lengths (5, 6, 7, 8, 9, and 10 amino acids), a solution of 10 (**b**), 9 (**c**), 8 (**d**), 7 (**e**), 6 (**f**), and 5 (**g**) amino acids long arginine peptides. Each histogram corresponding to a given peptide length (from (**b**–**g**)) exhibits a single *I*_b_/*I*_0_ population which also appears in the mixture histogram (**a**), allowing to discriminate and identify the peptides of different lengths in the mixture with a single amino acid resolution. The data were acquired in KCl 4 M HEPES 5 mM pH = 7.5 at −50 mV and at 20 °C

In order to ascertain whether the different populations observed in the *I*_b_/*I*_0_ histogram correspond to the different peptide lengths present in the arginine peptide mixture, experiments probing independently the interaction of arginine peptides of each individual length (5, 6, 7, 8, 9, 10 amino acids) with the aerolysin nanopore were performed under the same experimental conditions. The histograms of the relative blockade current *I*_b_/*I*_0_ values corresponding to each of the 6 different peptide lengths are shown in Fig. 2b–g. Each histogram shows an overwhelming majority population with a preferred *I*_b_/*I*_0_ value, corresponding to the peptide length used. The location of the majority population depends on the peptide length: the mean *I*_b_/*I*_0_ value of the majority population decreases as the peptide length increases. Comparing with the *I*_b_/*I*_0_ histogram of the peptide mixture (Fig. 2a) reveals that each majority population corresponding to a given peptide length coincides with one of the main populations observed in the mixture histogram. This enables to attribute an individual peptide length to each population observed in the mixture histogram, from 10 amino acids long arginine peptides for the deepest population (*I*_b_/*I*_0_ = 0.234) to 5 amino acids long arginine peptides for the shallowest population (*I*_b_/*I*_0_ = 0.631). This demonstrates the ability of the wild-type aerolysin nanopore to perform the size-discrimination of arginine peptides with a single amino acid resolution. Furthermore, measurements of the mean relative blockade current value and of the mean blockade duration corresponding to each individual peptide length are highly reproducible between independent experiments (Supplementary Fig. 2), highlighting the high-fidelity resolution power of the nanopore.

The effect of voltage on the interaction of the aerolysin nanopore with a mixture of arginine peptides of 6 different lengths (5 to 10 amino acids) is shown in Supplementary Figs. 3 and 4, for negative voltages ranging from −25 to −80 mV. For each voltage, the populations corresponding to the different peptide lengths in the mixture were identified from the *I*_b_/*I*_0_ histogram (Supplementary Fig. 4) and independently analyzed (see the Methods section). Regardless of the applied voltage, the mean blockade duration increases with the peptide length, up to two orders of magnitude between 5 amino acids long and 10 amino acids long peptides (Supplementary Fig. 3b). For each peptide length, the mean blockade duration depends non-monotonically on voltage, first increasing with voltage magnitude, reaching a maximum and then decreasing. The voltage corresponding to the maximum of the mean blockade duration depends only slightly on the peptide length, if at all, and is comprised between −35 and −40 mV. Note that the mean blockade duration corresponding to each peptide length are identical when measured whether in a peptide mixture experiment or in independent experiments for each peptide length.

The qualitative voltage dependence of the mean relative blockade current *I*_b_/*I*_0_ depends on the arginine peptide length (Supplementary Figs. 3a and 4): the mean *I*_b_/*I*_0_ slightly decreases with voltage magnitude for the longest peptides (10, 9, and 8 amino acids), is voltage-independent for intermediate peptide lengths (7 and 6 amino acids) and even slightly increases with voltage magnitude for the shortest peptides (5 amino acids). As shown in the *I*_b_/*I*_0_ histograms of the peptide mixture under different applied voltages (Supplementary Fig. 4), the size-discrimination of arginine peptides of different lengths with a single amino acid resolution is preserved in the whole voltage range explored, even at a voltage as low as −25 mV. Note that maximizing the mean blockade duration for each of the different peptide lengths (at ≈−40 mV) does not maximize the size-discrimination power of the nanopore. Interestingly, the different populations of the *I*_b_/*I*_0_ histogram of the peptide mixture become increasingly separated as the negative voltage magnitude increases. For voltage magnitudes greater than or equal to −50 mV (Supplementary Fig. 4 and Fig. 1d), an additional population with a mean *I*_b_/*I*_0_ ≈ 0.73 appeared. This additional population is the 7th population detected from an arginine peptide mixture of 6 different lengths, and is located in the short peptide part of the *I*_b_/*I*_0_ histogram. Observing this additional population was not possible at low voltages, probably due to the presence of a significant number of current fluctuations from the residual open pore current noise, with *I*_b_/*I*_0_ values between ≈0.7 and 0.8. As the voltage magnitude is increased, the signal-to-noise ratio is enhanced ($\frac{{I_0 - 5\sigma }}{{I_0}} = 0.83$ at −25 mV and 0.91 at −80 mV, *I*_0_ and *σ* being the mean open pore current and its standard deviation respectively, see the Methods section and Supplementary Fig. 8 for details); current fluctuations due to residual open pore current noise are shifted to greater *I*_b_/*I*_0_ values with increasing voltage magnitude, enabling the clear observation of the additional population whose identification is established in the next section.

### Detection and identification of peptide impurities

**Fig. 3:**
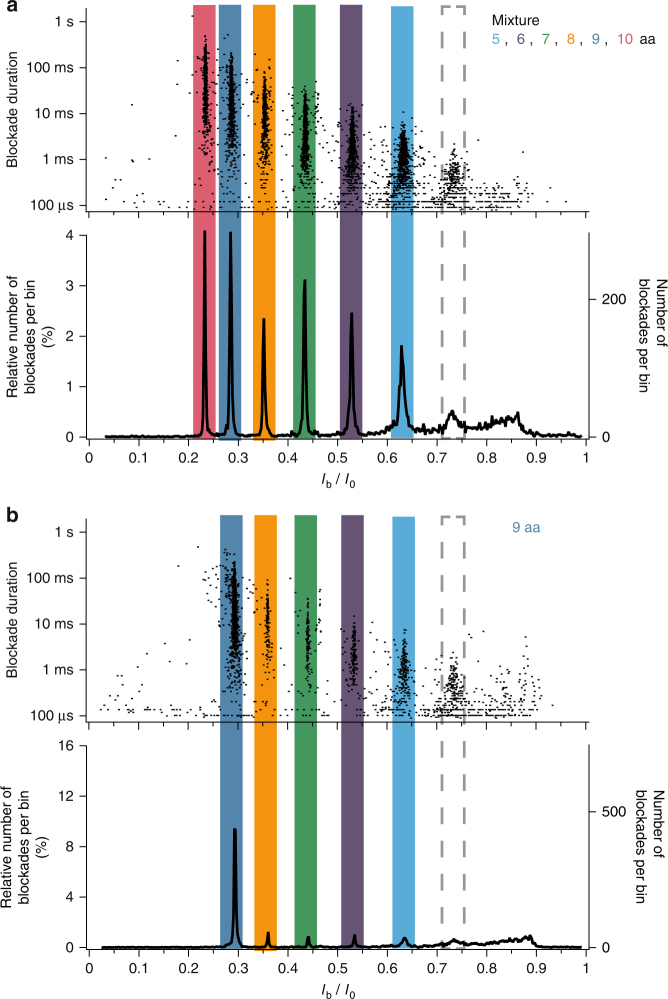
High sensitivity detection and identification of peptide impurities. **a** Interaction of aerolysin nanopore with an equimolar mixture of arginine peptides of different lengths (5, 6, 7, 8, 9, and 10 amino acids): (top) scatter plot of blockade duration versus relative blockade current *I*_b_/*I*_0_ (every dot corresponds to a single current blockade event); (bottom) histogram of the relative blockade current *I*_b_/*I*_0_ values. **b** Interaction of aerolysin nanopore with a solution of 9 amino acids long arginine peptides with a >98% purity in peptide length (purity value provided by the supplier): (top) scatter plot of blockade duration versus relative blockade current *I*_b_/*I*_0_ (every dot corresponds to a single current blockade event); (bottom) histogram of the relative blockade current *I*_b_/*I*_0_ values. In (**b**), in addition to the main population of 9 amino acids long peptides (dark blue), at least 4 minority populations of only a few dozens of molecules each are detected and identified as corresponding to 8 (yellow), 7 (green), 6 (purple), and 5 (light blue) amino acids long peptides. Another population (gray dashed rectangles) is detected at *I*_b_/*I*_0_ = 0.731 +/− 0.004 both in the peptide mixture (**a**) and in the 9 amino acids long peptides sample (**b**). The data were acquired in KCl 4 M HEPES 5 mM pH = 7.5 at −50 mV and at 20 °C

The histogram of the relative blockade current *I*_b_/*I*_0_ values and the scatter plot of blockade duration as a function of the relative blockade current are shown in Fig. 3b in the case of the interaction of a high-purity arginine peptide sample (>98% purity in peptide length) of given length (9 amino acids) with the aerolysin nanopore. As already observed in Fig. 2c, the *I*_b_/*I*_0_ histogram shows an overwhelming majority population corresponding to the 9 amino acids peptide length used. In addition to the majority population, at least four minority populations are detected. Each peak of the *I*_b_/*I*_0_ histogram corresponds to a distinct line-shaped cluster of dots in the scatter plot of blockade duration as a function of the relative blockade current, showing the different populations, and in particular the minority populations, even more clearly. Comparing with the case of the interaction of an equimolar mixture of arginine peptides of 6 different lengths (5 to 10 amino acids) with the aerolysin nanopore (Fig. 3a) reveals that the minority populations detected in the high-purity 9 amino acids long arginine peptide sample correspond to arginine peptide fragments whose length can be identified (5, 6, 7, 8 amino acids). Interestingly, quantities as small as a few dozens of molecules are detected for each peptide fragment length (see the right axis of the *I*_b_/*I*_0_ histogram in Fig. 3b), revealing the high sensitivity of the nanopore system. In contrast, the purity analysis provided by the peptide supplier using conventional techniques (High Purity Liquid Chromatography, HPLC, and Mass Spectrometry, MS) did not identify these peptide fragments, including them among general impurities (Supplementary Fig. 5). This demonstrates that the aerolysin nanopore enables the detection, identification and quantification of the peptide impurities present in a high-purity peptide sample with a higher sensitivity than the conventional purity analysis techniques (HPLC and MS).

**Fig. 4:**
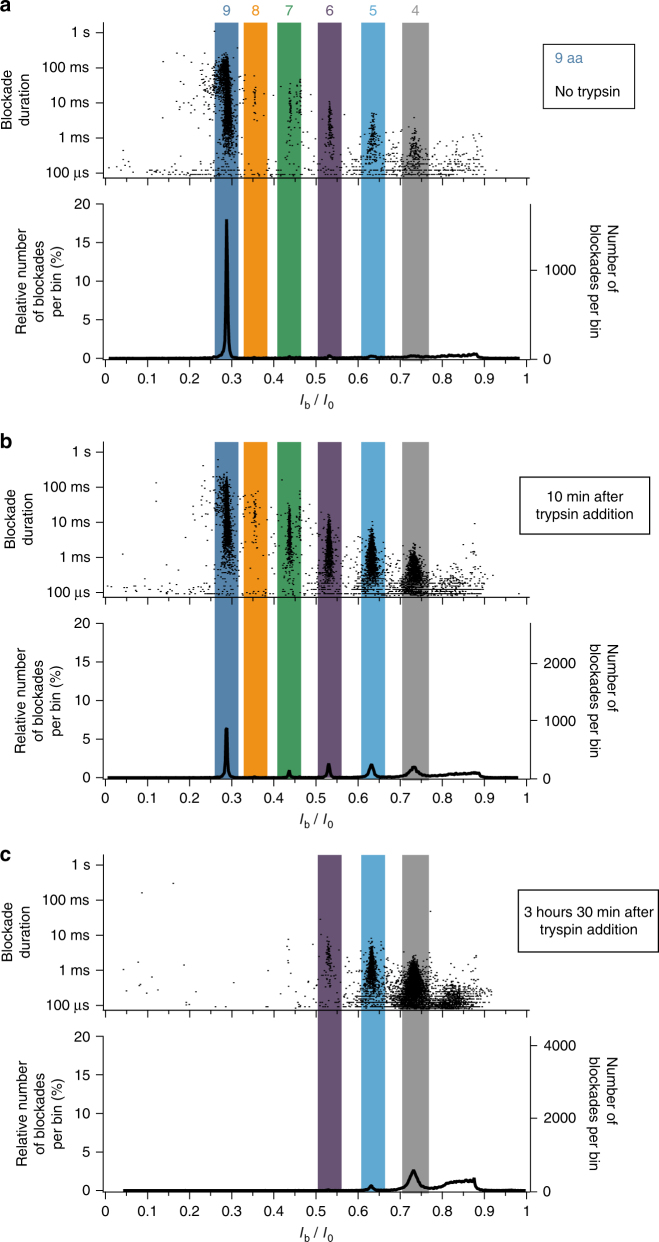
Real-time monitoring of the enzymatic degradation of an arginine peptide sample. Scatter plot of blockade duration versus relative blockade current *I*_b_/*I*_0_ (top of each subfigure) and histogram of the relative blockade current *I*_b_/*I*_0_ values (bottom of each subfigure) in the case of the interaction of aerolysin nanopore with a solution of 9 amino acids long arginine peptides: **a** in absence of trypsin enzyme, **b** 10 min after trypsin addition and (**c**) 3 h 30 min after trypsin addition. Colored numbers on top of subfigure (**a**) indicate the number of amino acids corresponding to each blockade population. In absence of trypsin, the overwhelming majority of blockades corresponds to 9 amino acids long peptides. Only 10 min after trypsin addition, a significant (≈3 folds) decrease of the proportion of blockades corresponding to 9 amino acids long peptides is observed, in favor of a significant increase of the proportion of blockades corresponding to shorter peptides, indicating the trypsin cleavage of long peptides into shorter ones. In particular, the increase of the proportion of blockades with *I*_b_/*I*_0_ ≈ 0.73 (gray) confirms that this population corresponds to 4 amino acids long peptides. After 3 h 30 min of trypsin activity, the 9, 8, and 7 amino acids long peptides populations have disappeared. The data were acquired in KCl 4 M HEPES 5 mM pH = 7.5 at −50 mV and at 20 °C

As already observed in the arginine peptide mixture analysis for negative voltage magnitudes greater than or equal to −50 mV (Fig. 1d and Supplementary Fig. 4), an additional and unidentified population exists in the analysis of the high-purity 9 amino acids long arginine peptide sample, located on the short-peptides side (*I*_b_/*I*_0_ ≈ 0.73) (Fig. 3b). In order to ascertain whether this additional population was an artifact from the experimental setup or corresponded to analytes in solution (*e.g*. 4 amino acids long arginine peptides present as impurities), experiments monitoring the real-time enzymatic degradation of arginine peptides were performed. The histograms of the relative blockade current *I*_b_/*I*_0_ values and the scatter plots of blockade duration as a function of the relative blockade current are shown in Fig. 4 in the case of the interaction of a 9 amino acids long arginine peptide sample with the aerolysin nanopore in absence of trypsin enzyme (Fig. 4a), 10 min after trypsin addition (Fig. 4b) and 3 h 30 min after trypsin addition (Fig. 4c). As time increases, the number of current blockades corresponding to long peptides decreases while the number of current blockades corresponding to shorter peptides increases, indicating the enzymatic cleavage of long peptides into shorter ones. In particular, the population located at *I*_b_/*I*_0_ ≈ 0.73 increases with time, revealing that this population, observed here and in previous Figures (Figs. 1d and 3b and Supplementary Fig. 4) corresponds to 4 amino acids long arginine peptides. Furthermore, the population located at *I*_b_/*I*_0_ ≈ 0.84 increases with time, suggesting that this population could correpond to 3 amino acids long arginine peptides. This is confirmed by an independent experiment using a solution of 3 amino acids long arginine peptides (Supplementary Fig. 10).

### Discrimination between lysine and arginine peptides

In order to ascertain whether the above-described method could be used with other peptides than arginine peptides, experiments probing the interaction of lysine peptides with the aerolysin nanopore were performed.

**Fig. 5:**
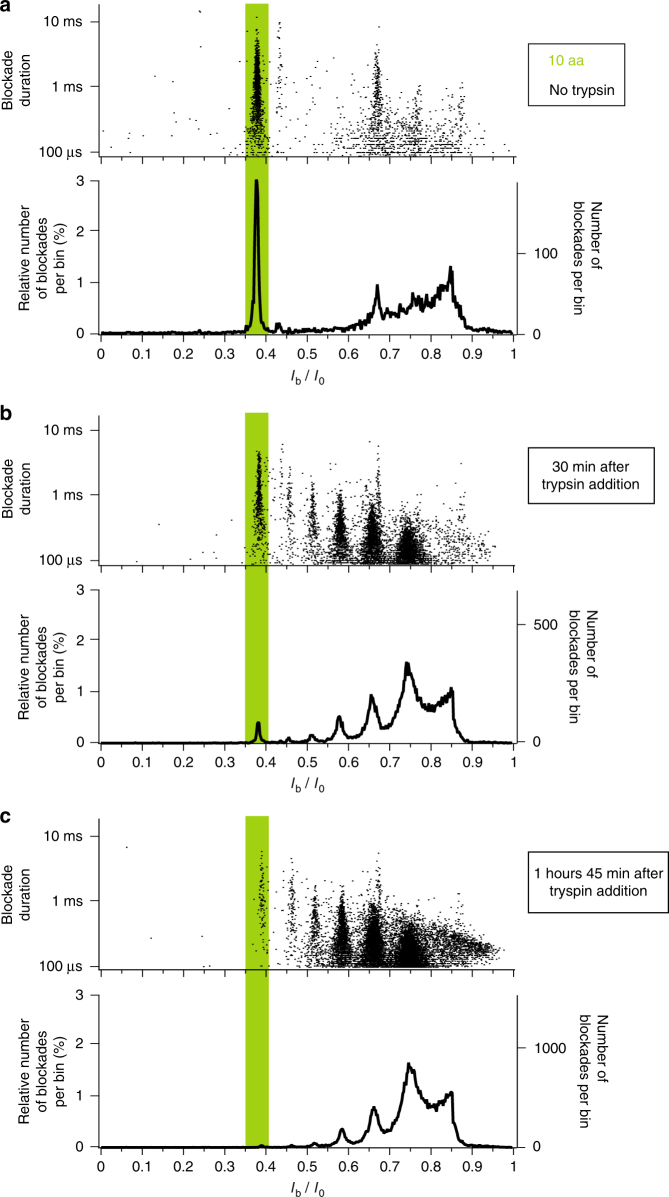
Real-time monitoring of the enzymatic degradation of a lysine peptide sample. Scatter plot of blockade duration versus relative blockade current *I*_b_/*I*_0_ (top of each subfigure) and histogram of the relative blockade current *I*_b_/*I*_0_ values (bottom of each subfigure) in the case of the interaction of aerolysin nanopore with a solution of 10 amino acids long lysine peptides: **a** in absence of trypsin enzyme, (**b**) 30 min after trypsin addition, and (**c**) 1 h 45 min after trypsin addition. In absence of trypsin, the majority of blockades corresponds to 10 amino acids long peptides. After trypsin addition, a significant decrease of the proportion of blockades corresponding to 10 amino acids long peptides is observed, in favor of a significant increase of the proportion of blockades corresponding to shorter peptides, indicating the trypsin cleavage of long peptides into shorter ones. The data were acquired in KCl 4 M HEPES 5 mM pH = 7.5 at −50 mV and at 20 °C

The histograms of the relative blockade current *I*_b_/*I*_0_ values and the scatter plots of blockade duration as a function of the relative blockade current are shown in Fig. 5 in the case of the interaction of a high-purity (>98%) 10 amino acids long lysine peptide sample with the aerolysin nanopore in absence of trypsin enzyme (Fig. 5a), 30 min after trypsin addition (Fig. 5b) and 1 h 45 min after trypsin addition (Fig. 5c). Before trypsin addition (Fig. 5a), the *I*_b_/*I*_0_ histogram shows an overwhelming majority population, corresponding to the 10 amino acids lysine peptide length used, and additional minority populations, as in the case of arginine peptides. Moreover, as time increases after trypsin addition (Fig. 5b,c), the number of current blockades corresponding to long lysine peptides decreases while the number of current blockades corresponding to shorter lysine peptides increases, indicating the enzymatic cleavage of long lysine peptides into shorter ones, as in the case of arginine peptides. This observation is an important indicator of the ability of the wild-type aerolysin nanopore to perform the size-discrimination of lysine peptides.

**Fig. 6:**
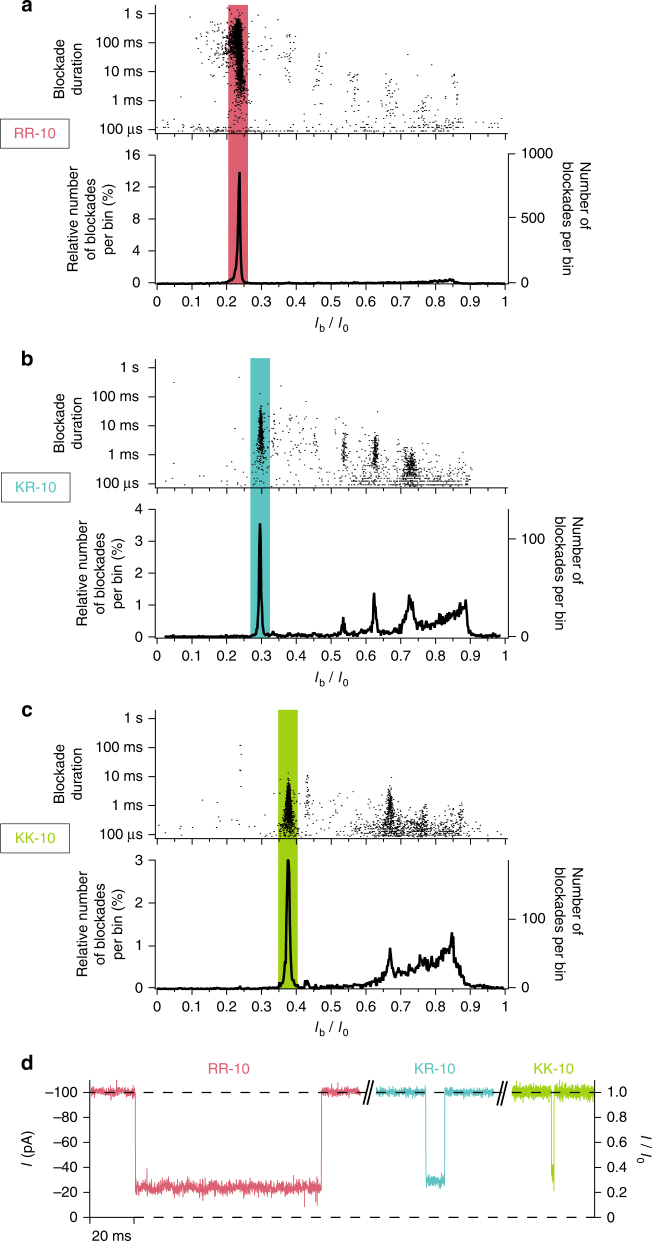
Discrimination of 10 amino acids long peptides of different sequences. **a**–**c** Scatter plot of blockade duration versus relative blockade current *I*_b_/*I*_0_ (top of each subfigure) and histogram of the relative blockade current *I*_b_/*I*_0_ values (bottom of each subfigure) in the case of the interaction of aerolysin nanopore with: (**a**) a solution of 10 amino acids long arginine homopeptides (RRRRRRRRRR, named RR-10), (**b**) a solution of 10 amino acids long lysine/arginine heteropeptides (KKKKKRRRRR, named KR-10) and (**c**) a solution of 10 amino acids long lysine homopeptides (KKKKKKKKKK, named KK-10). Each histogram corresponding to a given peptide sequence exhibits a main *I*_b_/*I*_0_ population clearly distinct from the other peptide sequences, allowing to discriminate between peptides of same length but of different sequences. **d** Typical current blockades in the case of the interaction of aerolysin nanopore with (from left to right) a solution of RR-10 peptides, with a solution of KR-10 peptides and with a solution KK-10 peptides. The left axis indicates the current *I* values (negative current values under a negative applied voltage) and the right axis indicates the relative current *I*/*I*_0_ values. For each peptide sequence, the typical current blockade has a relative blockade current *I*_b_/*I*_0_ value and a blockade duration respectively equal to the mean *I*_b_/*I*_0_ value and to the mean blockade duration of the main population of the histograms shown in (**a**–**c**) (see also Supplementary Fig. 7). The data were acquired in KCl 4 M HEPES 5 mM pH = 7.5 at −50 mV and at 20 °C

The histograms of the relative blockade current *I*_b_/*I*_0_ values and the scatter plots of blockade duration as a function of the relative blockade current are shown in Fig. 6 in the case of the interaction of the aerolysin nanopore with a high-purity (>98%) sample of 10 amino acids long arginine peptides (Fig. 6a) and of 10 amino acids long lysine peptides (Fig. 6c). Interestingly, the observed majority population clearly differs between the two different peptides, both in term of mean *I*_b_/*I*_0_ value and in term of mean blockade duration. The mean *I*_b_/*I*_0_ value is 0.236 +/− 0.003 in the case of arginine and 0.377 +/− 0.004 in the case of lysine, while the mean blockade duration decreases by almost two orders of magnitude from nearly 100 ms in the case of arginine to nearly 1 ms in the case of lysine (see also Supplementary Fig. 7). This reveals that our method enables the discrimination between arginine and lysine homopeptides of same length, even while arginine and lysine amino acids have the same electrical charge and very similar chemical structures.

Moreover, experiments probing the interaction of the aerolysin nanopore with a high-purity (>98%) sample of 10 amino acids long heteropeptides formed by a sequence of 5 lysine amino acids coupled to 5 arginine amino acids (Fig. 6b) reveal that this peptide can also be discriminated from 10 amino acids long arginine peptides and from 10 amino acids long lysine peptides (Fig. 6a–c and Supplementary Fig. 7), highlighting the high-sensitivity of our method. In the case of 10 amino acids long lysine/argine heteropeptides, the mean *I*_b_/*I*_0_ value is 0.297 +/− 0.003 and the mean blockade duration is nearly 10 ms.

## Discussion

The high resolution of aerolysin pore for the highly-sensitive analysis of short peptides is most likely attributed to its geometry, to its highly charged pore wall and to the high affinity between aerolysin and peptides (see Supplementary Fig. 6). Nanopore-based single amino acid size-discrimination of several peptide lengths mixed in solution that differ by a single amino acid is subject to four conditions: i) the whole peptide must be entirely fitted en bloc inside the vicinity of the pore, ii) all amino acids must contribute individually to the current blockades, iii) a high size-sensitivity of the pore conductance change and iv) a high affinity between the pore and the peptide (the life time of the peptide/pore interaction must exceed 2 times the rise time of the bandwidth). In our experiments, observing different residual blockade currents in the case of peptides differing by only one amino acid indicates that each amino acid contributes individually to the current blockade, corresponding to a coil or to a compact conformation of the peptide in the nanopore and not to a rod-like conformation of the peptide. In the hypothesis of a rod-like conformation of the peptide, it is expected that current blockades would only weakly, if at all, depend on the peptide length. A coil or compact conformation of the peptide is not surprising in the high salt conditions of our experiments and considering the short persistence length of peptides (≃2 amino acids)^69^. Concerning peptides longer than 10 amino acids, experiments performed with 12 amino acids long arginine homopeptides (Supplementary Fig. 11) display a distinct preferential residual blockade current population corresponding to this peptide length, as observed for shorter peptides. Our perspectives are rather turned towards the analysis of short peptides, as short as a single amino acid, which is of particular importance towards peptide/protein sequencing. Furthermore, short peptides are widely used by cells for their communications in normal and pathologic conditions. These short peptides can be found either in the intracellular or extracellular compartments and some of them are secreted or released into extracellular vesicles, assuming functions both inside and outside the cell. Otherwise, for industrial applications, our proof-of-concept method could be extended to longer homopeptides of different chemical nature by using larger biological nanopores such as ClyA nanopore^28^. Under appropriate experimental conditions, our proof-of-concept method could be also extended to the size-discrimination and purity analysis of other short homopeptides and heteropeptides. A possible way out would be to use low^38^ or high pH conditions, where polypeptides might be uniformly charged, or not charged at all.

Ultra-fast protein recognition of peptide and protein with a nanopore is another challenge that already started. The first attempts focused on the detection and discrimination of native proteins either outside^7,22,23,56,59^ or inside^21,29,35,38,39^ the nanopore. Then an approach towards protein sequencing was to unfold the native protein and to try to read the polypeptide sequence into the nanopore during the translocation process. An interesting work demonstrated protein unfolding and translocation through a biological nanopore using an unfoldase molecular motor (ClpX variant)^27^. The ionic current trace during protein translocation did not allow to detect single amino-acid differences along the polypeptide sequence but showed electrical signal differences according to various protein domains with a maximum resolution of ≈663 amino acids^70^. Up to now, unfolded protein transport through a biological nanopore was too fast to read the protein sequence^18,48^. Recently a subnanometre diameter pore was manufactured, from a silicon nitride membrane, to indirectly read the primary sequence of an unfolded protein with a resolution of blocks of four amino acids, based on a theoretical excluded volume model^71^. So far, solid-state nanopores still suffer from a poorly reproducible manufacturing process and from insufficient wetting surface control. Here, in contrast, production of recombinant biological nanopores is standardized and highly reproducible. The electrical signal measurements to detect the size of short peptides with a single amino acid resolution and to discriminate between different chemical natures of homo-and heteropeptides are also highly reproducible. The high current blockade depth resolution -single amino acid- combined with the high temporal resolution –up to 10 ms/amino acid, i.e., a 3 orders of magnitude improvement as compared to previous published data^18^– of our system pave the road for the construction of a sequence library of all the different amino acids. As a first perspective, coupling the detection and identification of individual amino acids of different chemical nature either with the controlled translocation of an unfolded peptide/protein or with the sequential degradation of a peptide/protein near the nanopore entrance, would provide a new tool for peptide/protein sequencing. As a second perspective, combining the single-molecule sensitivity of the nanopore technology with a nanofluidic device guiding a single cell to the nanopore entrance would allow to quantify very small quantities of peptide biomarkers in a single cell. For instance, recently, Maglia and co-workers analyzed model peptide and protein biomarkers through FraC nanopores^38^. This might lead to important advances in the field of single-molecule proteomics. Our results and those of more recent works^37–39,59,72^ pave the road to analyse realistic samples, such as those from blood stream, serum, biopsies, or lysate extracts.

## Methods

### Synthesis of the aerolysin nanopore

The aerolysin nanopore is a pore-forming toxin from *Aeromonas hydrophila* which forms an heptameric *β*-barrel pore assembly in cell membranes^73^. The atomistic structure of aerolysin was recently defined^74^. It has an inner pore diameter of 1.0–1.7 nm and has a high number of charged residues (91 charges) pointing into the pore lumen^74^. Recombinant wild-type pro-aerolysin was synthetized using the following procedure. We transformed *Escherichia coli* BL21 strain with pET22b-proAL plasmid containing pro-aerolysin sequence. This plasmid allowed the induction of pro-aerolysin production with IPTG (1 mM final concentration) and the periplasmic localization of the recombinant protein. The periplasm was extracted with an osmotic shock and pro-aerolysin was furthermore purified by affinity chromatography using the C-terminal his-tag of the recombinant protein (His SpinTrap minicolumns, GE Healthcare Life Science). Pro-aerolysin binding to the Ni-Sepharose was made with 100 mM Tris-HCl pH 7.4 and 50 mM imidazole, and after three washing steps, elution was made with 100 mM Tris-HCl pH 7.4 and 500 mM imidazole. The recombinant pro-aerolysin purity was determined by SDS-polyacrylamide gel electrophoresis and Coomassie bleu staining, to 99 ± 1% (w/w). Concentration was calculated by absorbance at 280 nm. The propeptides of pro-aerolysin monomers were stored at 4 °C at a final concentration of 0.1 g/L.

### Nanopore recordings

A classical vertical lipid bilayer setup (Warner Instruments, Hamden, CT, USA or Elements, Cesena (FC), Italy) was used for all the experiments. The lipid bilayer was formed by painting and thinning a film of diphytanoyl-phosphocholine (Avanti Polar Lipids, Alabaster, AL, USA) dissolved in decane (10 mg/mL) over a 150 μm-wide aperture separating the *cis* and *trans* chambers. Both chambers were filled with 1 mL of 4 M KCl solution buffered with 5 mM HEPES and set to pH = 7.5. Two Ag/AgCl electrodes were used to apply a transmembrane voltage and to measure the transmembrane ionic current. The *cis*-chamber was at voltage ground.

In the case of the Warner Instruments setup, single-channel current recordings were performed using an Axopatch 200B patch-clamp amplifier (Molecular Devices, Sunnyvale, CA, USA) in the whole-cell mode with a CV-203BU headstage. The signal was filtered using an internal 4-pole Bessel filter at a cut-off frequency of 5 kHz. Data were acquired with a DigiData 1440A AD-converter (Molecular Devices) controlled by the Clampex 10.2 software (Molecular Devices). Current recordings at constant voltage were performed at a sampling rate of 250 kHz. Current versus voltage recordings were performed from −100 to +100 mV during 4 s and at a sampling rate of 5 kHz. A Peltier device controlled by a bipolar temperature controller (CL-100, Warner Instruments) allowed to set the temperature of the solution via a water circuit (LCS-1, Warner Instruments) around the chambers. The temperature was set to 20.0 ± 2 °C for all the experiments.

In the case of the Elements setup, single-channel current recordings were performed using an eONE amplifier (Elements, Cesena (FC), Italy). The signal was filtered at a cut-off frequency of 5 kHz. Data were acquired using the Elements Data Reader software (Elements, Cesena (FC), Italy), at a sampling rate of 200 kHz.

Recombinant wild-type pro-aerolysin was activated by trypsin digestion (0.6 μM trypsin final concentration) during 15 min at room temperature to eliminate the pro-peptide sequence. After the formation of the lipid bilayer, activated aerolysin was added to the *cis*-chamber at ≈1 nM final concentration. The insertion of a single nanopore in the lipid bilayer was identified as a step-jump of the transmembrane ionic current.

High-purity (>98%) arginine homopeptide samples (ProteoGenix, Schiltigheim, France) of different lengths (3, 5, 6, 7, 8, 9, 10, 12 amino acids) were used. Peptides were dissolved in aqueous 5 mM HEPES pH = 7.5 buffer. In the case of current recordings at constant voltage, peptides were added to the *cis*-chamber, either as a 1 μM equimolar mixture of peptides of different lengths or as a 1 μM solution of peptides of a given length. In the case of current versus voltage recordings, 10 amino acids long peptides were added either to the *trans*-chamber at a 6 μM concentration, or to the *cis*-chamber at a 1.2 μM concentration (Supplementary Fig. 1). In each case, after peptide addition, a ≈2 mL mixing of the volume of the chamber corresponding to the side of peptide addition was performed, and a ≈10 min waiting time was observed in order to ensure an homogeneous peptide concentration in the chamber.

The enzymatic degradation of arginine homopeptides was performed by adding trypsin at 25 nM final concentration to a 4.4 μM solution of 9 amino acids long peptides. Ten minutes current recordings were performed every 10 min after trypsin addition, during 3 h 30 min.

High-purity (>98%) lysine homopeptide samples (ProteoGenix, Schiltigheim, France) of 10 amino acids were used. Peptides were dissolved in aqueous 5 mM HEPES pH = 7.5 buffer. Peptides were added to the *cis*-chamber as a 213 μM solution of peptides. After peptide addition, a ≈2 mL mixing of the volume of the chamber corresponding to the side of peptide addition was performed, and a ≈10 min waiting time was observed in order to ensure an homogeneous peptide concentration in the chamber.

The enzymatic degradation of lysine homopeptides was performed by adding trypsin at 266 nM final concentration to a 213 μM solution of 10 amino acids long peptides. 5 min current recordings were performed every 5 min after trypsin addition, during 1 h 45 min.

High-purity (>98%) lysine/arginine heteropeptide samples (ProteoGenix, Schiltigheim, France) of 10 amino acids were used. Peptides were dissolved in aqueous 5 mM HEPES pH = 7.5 buffer. Peptides were added to the *cis*-chamber as a 1 μM solution of peptides. After peptide addition, a ≈2 mL mixing of the volume of the chamber corresponding to the side of peptide addition was performed, and a ≈10 min waiting time was observed in order to ensure an homogeneous peptide concentration in the chamber.

### Data treatment

The data treatment was performed using the Igor Pro 6.12A software (WaveMetrics, Portland, OR, USA) with homemade procedures. The treatment was based on a statistical analysis of a least 2000 blockade events for each current trace. A two-thresholds method was used to detect peptide-induced current blockades (Supplementary Fig. 8). A first threshold th_1_ = *I*_0_ − 4*σ* was used to define every possible blockade event, where *I*_0_ and *σ* are respectively the mean open pore current and its standard deviation obtained from a gaussian fit of the open pore current distribution. A possible blockade event begins when the pore current decreases below th_1_ and ends when the pore current increases above th_1_. This first threshold allows to eliminate the overwhelming part of the open pore current noisy fluctuations. If the mean blockade current value *I*_b_ during the event is inferior to a second threshold th_2_ = *I*_0_ − 5*σ*, then the event is considered as a peptide-induced blockade event.

Histograms of the relative blockade current *I*_b_/*I*_0_ values were obtained using a bin width of 0.002. For each peptide length, the mean relative blockade current value $\left\langle {I_{\mathrm{b}}{\mathrm{/}}I_0} \right\rangle _i$ (*i* = 5, 6, 7, 8, 9, or 10 amino acids long peptides) corresponds to the mean value of a gaussian fit of the *I*_b_/*I*_0_ peak corresponding to this peptide length. The error bar of each $\left\langle {I_{\mathrm{b}}{\mathrm{/}}I_0} \right\rangle _i$ corresponds to the standard deviation *σ*_*i*_ of the gaussian fit of the peak. The current blockades corresponding to each peptide length were identified as the current blockades with *I*_b_/*I*_0_ values comprised between $\left\langle {I_{\mathrm{b}}{\mathrm{/}}I_0} \right\rangle _i - 3\sigma _i$ and $\left\langle {I_{\mathrm{b}}{\mathrm{/}}I_0} \right\rangle _i + 3\sigma _i$. From the current blockades corresponding to each peptide length, the mean blockade duration and frequency were calculated for each peptide length. The mean blockade duration is the mean value of: the calculated mean value of blockade durations, the mean blockade duration obtained from a single exponential fit of the blockade durations distribution and the mean blockade duration obtained from a single exponential fit of the cumulative blockade durations distribution (Supplementary Fig. 9). The error bar of the mean blockade duration is the maximal value among: the sampling time interval of the acquisition system (4 μs at 250 kHz sampling rate), the dispersion of the mean blockade durations obtained from the 3 methods above and the standard deviation of the fit coefficients from the data analysis software. The same procedure applied to the inter-blockade durations provided the mean inter-blockade duration, and thus the mean blockade frequency, and its error bar for each peptide length.

The results were reproduced with a high fidelity from at least 4 independent experiments (Supplementary Fig. 4). The results presented in this article correspond to individual experiments.

### Data availability

The authors declare that the data supporting the findings of this study are available within the article and its Supplementary Information files or from the corresponding authors upon reasonable request.

## Electronic supplementary material

Supplementary Information
