# Supplementary material for: Identification of single amino acid differences in uniformly charged homopolymeric peptides with aerolysin nanopore
Source: Nat Commun. 2018 Mar 6;9:966. doi: 10.1038/s41467-018-03418-2 (PMC5840376; doi:10.1038/s41467-018-03418-2)
Supplement: Supplementary file 1 — Supplementary Information [file 41467_2018_3418_MOESM1_ESM.pdf]

Identification of single amino acid differences in uniformly charged homopolymeric peptides with aerolysin nanopore

Fabien Piguet[1,⋆,†], Hadjer Ouldali[1,†], Manuela Pastoriza-Gallego[1], Philippe Manivet[2,3], Juan Pelta[4,⋆] and Abdelghani Oukhaled[1,⋆]

[1] LAMBE UMR 8587, Université de Cergy-Pontoise, 95300 Pontoise, France
[2] APHP, Centre de Ressources Biologiques BB-0033-00064, Plateforme de Bio-Pathologie et de Technologies Innovantes en santé, Hôpital Lariboisière, 75010 Paris, France
[3] INSERM UMR-S942, Hôpital Lariboisière, 75010 Paris, France
[4] LAMBE UMR 8587, Université d'Evry-Val-d'Essonne, 91000 Evry, France

†: contributed equally

⋆ e-mail: fabien.piguet@u-cergy.fr
⋆ e-mail: juan.pelta@univ-evry.fr
⋆ e-mail: abdelghani.oukhaled@u-cergy.fr

Supplementary Information

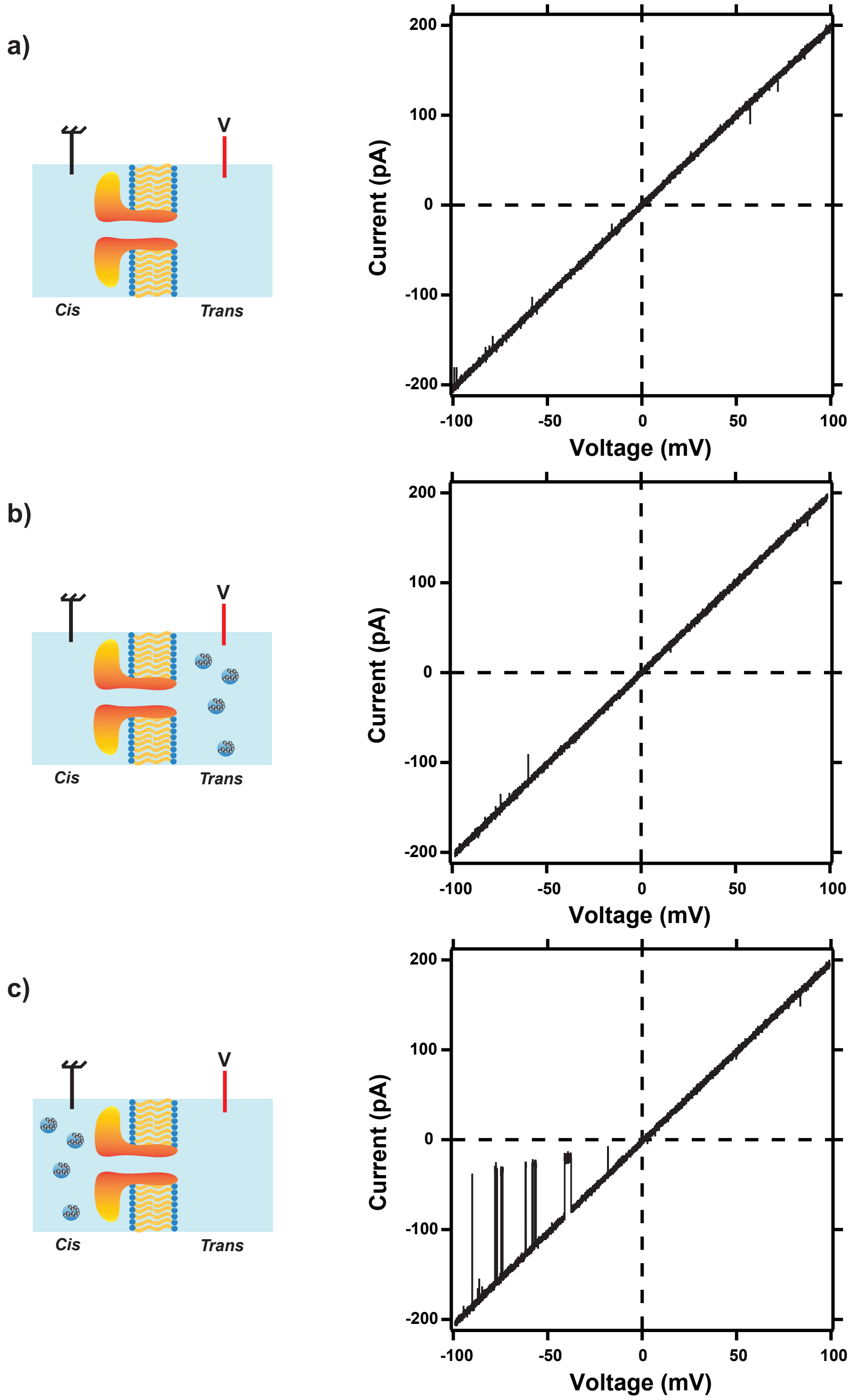

Supplementary Figure 1: **Detection of arginine peptides with a wild-type aerolysin nanopore according to the nanopore side from where peptides were added and to the voltage polarity.** Current *versus* voltage curves through the aerolysin nanopore: (a) in absence of analytes, (b) in presence of 6μM of 10 amino acids long arginine peptides added from the *trans*-side of the lipid bilayer, (c) in presence of 1.2μM of 10 amino acids long arginine peptides added from the *cis*-side of the lipid bilayer. The data were acquired in KCl 4M HEPES 5mM pH=7.5 at 20°C and the duration of each current-voltage curve was 4 seconds.

a)

b)

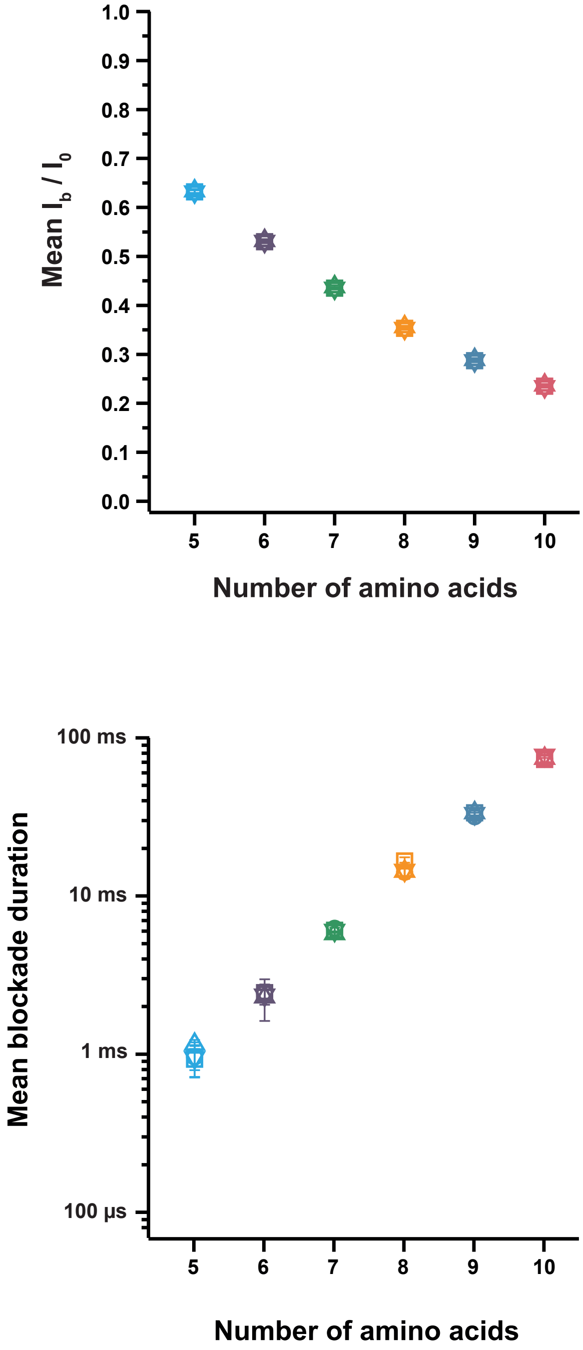

Supplementary Figure 2: **Reproducibility of the experimental results.** Superimposed results from 4 independent experiments (○, □, △ and ▽ symbols) in the case of the interaction of aerolysin nanopore with an equimolar mixture of arginine peptides of different lengths (5, 6, 7, 8, 9 and 10 amino acids): (a) mean relative blockade current I_b/I_0 and (b) mean blockade duration as a function of the number of amino acids. The data were acquired in KCl 4M HEPES 5mM pH=7.5 at -50 mV and at 20°C.

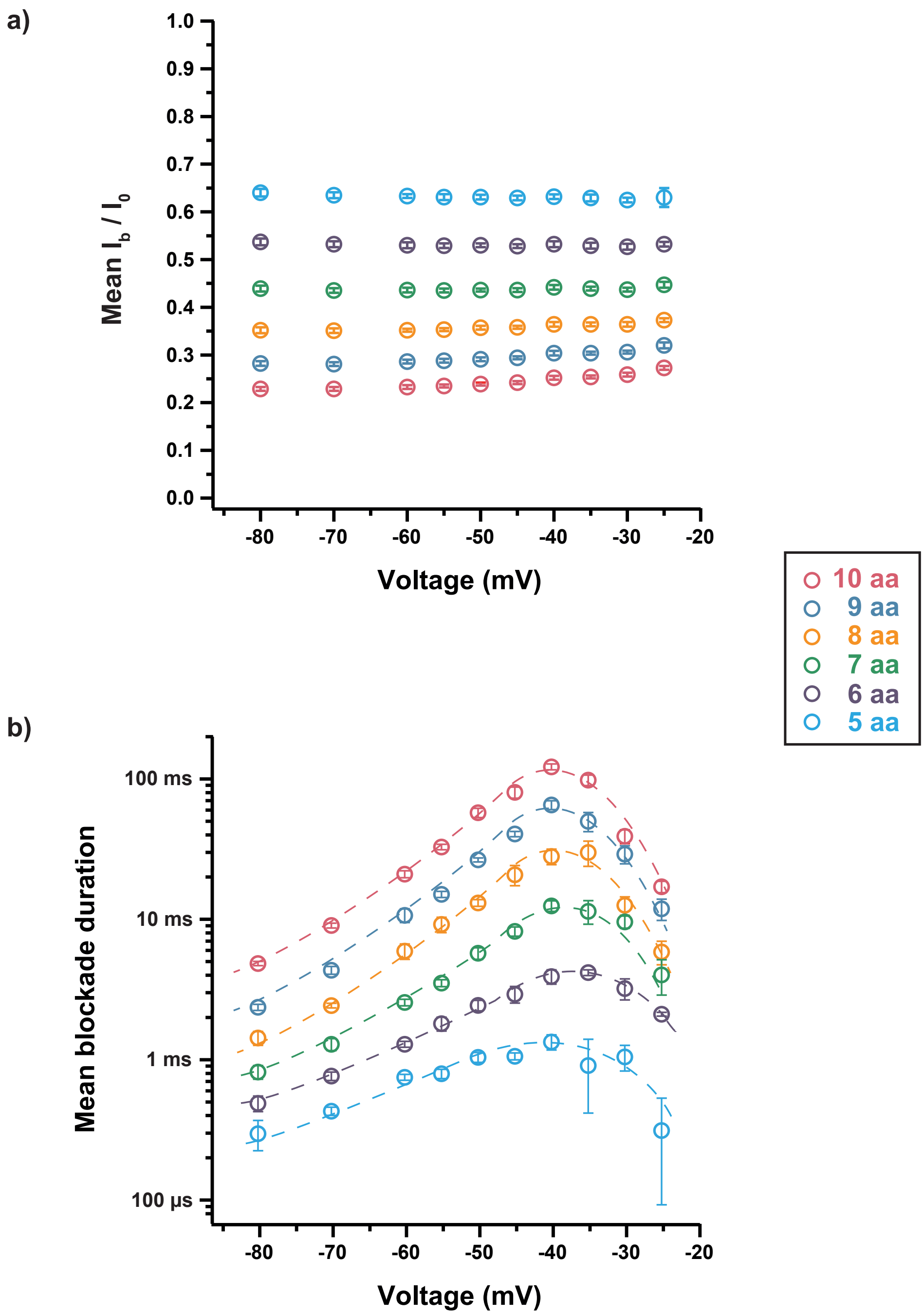

Supplementary Figure 3: **Effect of voltage on the interaction of aerolysin nanopore with a mixture of arginine peptides of different lengths.** (a) Mean relative blockade current I_b/I_0 *versus* voltage and (b) mean blockade duration *versus* voltage for each peptide length present in the mixture (10 (red), 9 (dark blue), 8 (yellow), 7 (green), 6 (purple) and 5 (light blue) amino acids). Dashed lines are guides to the eye. The data were acquired in KCl 4M HEPES 5mM pH=7.5 at 20°C.

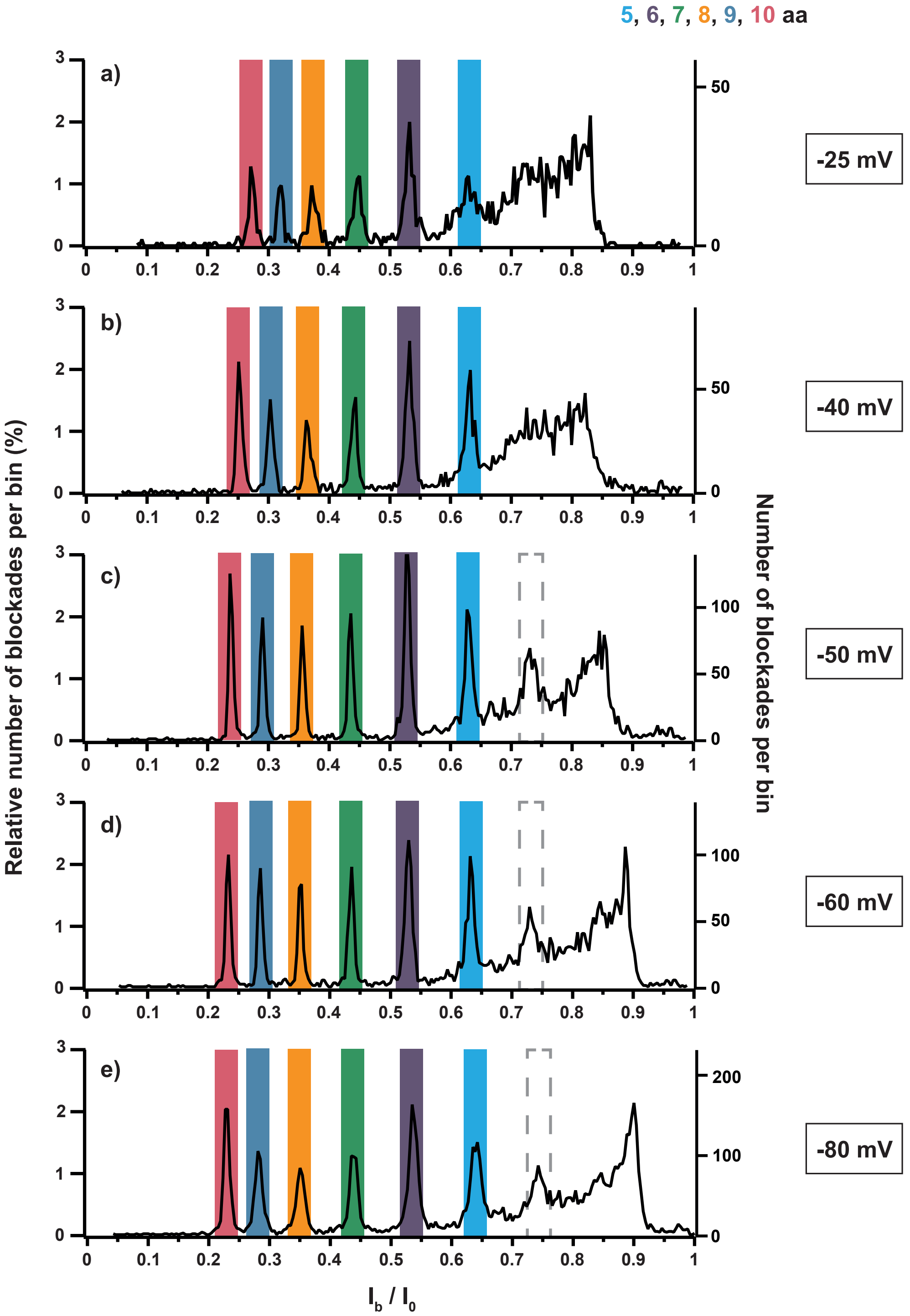

Supplementary Figure 4: **Effect of voltage on the discrimination of arginine peptides of different lengths.** Histograms of the relative blockade current I_b/I_0 values in the case of the interaction of aerolysin nanopore with an equimolar mixture of arginine peptides of different lengths (5, 6, 7, 8, 9 and 10 amino acids) at -25 mV (a), -40 mV (b), -50 mV (c), -60 mV (d) and -80 mV (e). The data were acquired in KCl 4M HEPES 5mM pH=7.5 at 20°C. For voltage magnitudes smaller than -50 mV (a and b) 6 different populations, corresponding to 6 different peptide lengths from 5 to 10 amino acids, are observed. For voltage magnitudes greater than or equal to -50 mV (c, d and e), a 7th population (dashed grey rectangles) is observed.

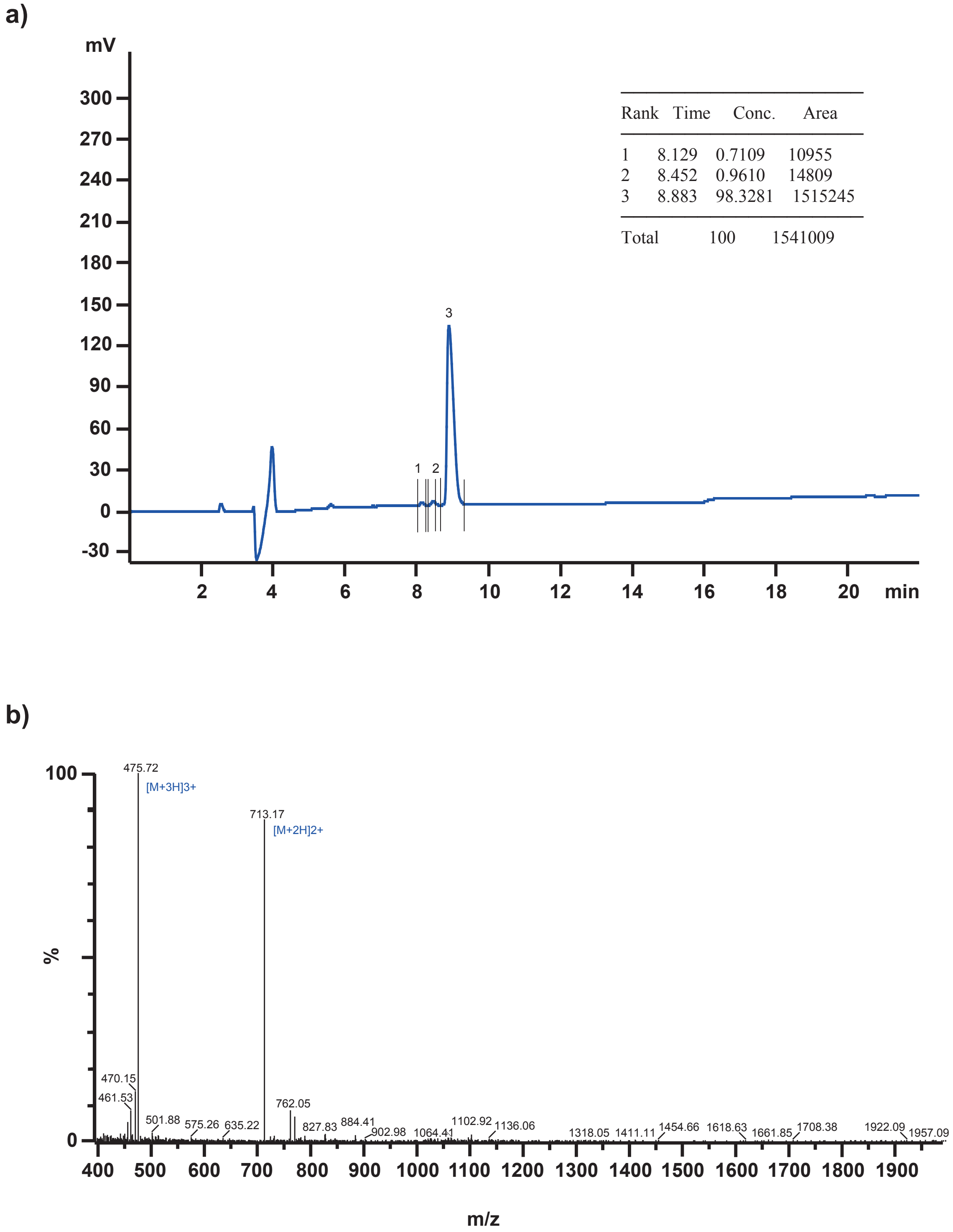

Rank	Time	Conc.	Area
1	8.129	0.7109	10955
2	8.452	0.9610	14809
3	8.883	98.3281	1515245
Total		100	1541009

Supplementary Figure 5: **Supplier-provided purity analysis of a high purity (> 98%) 9 amino acids long arginine peptide sample.** (a) High Performance Liquid Chromatography (HPLC) analysis. (b) Mass Spectrometry (MS) analysis.

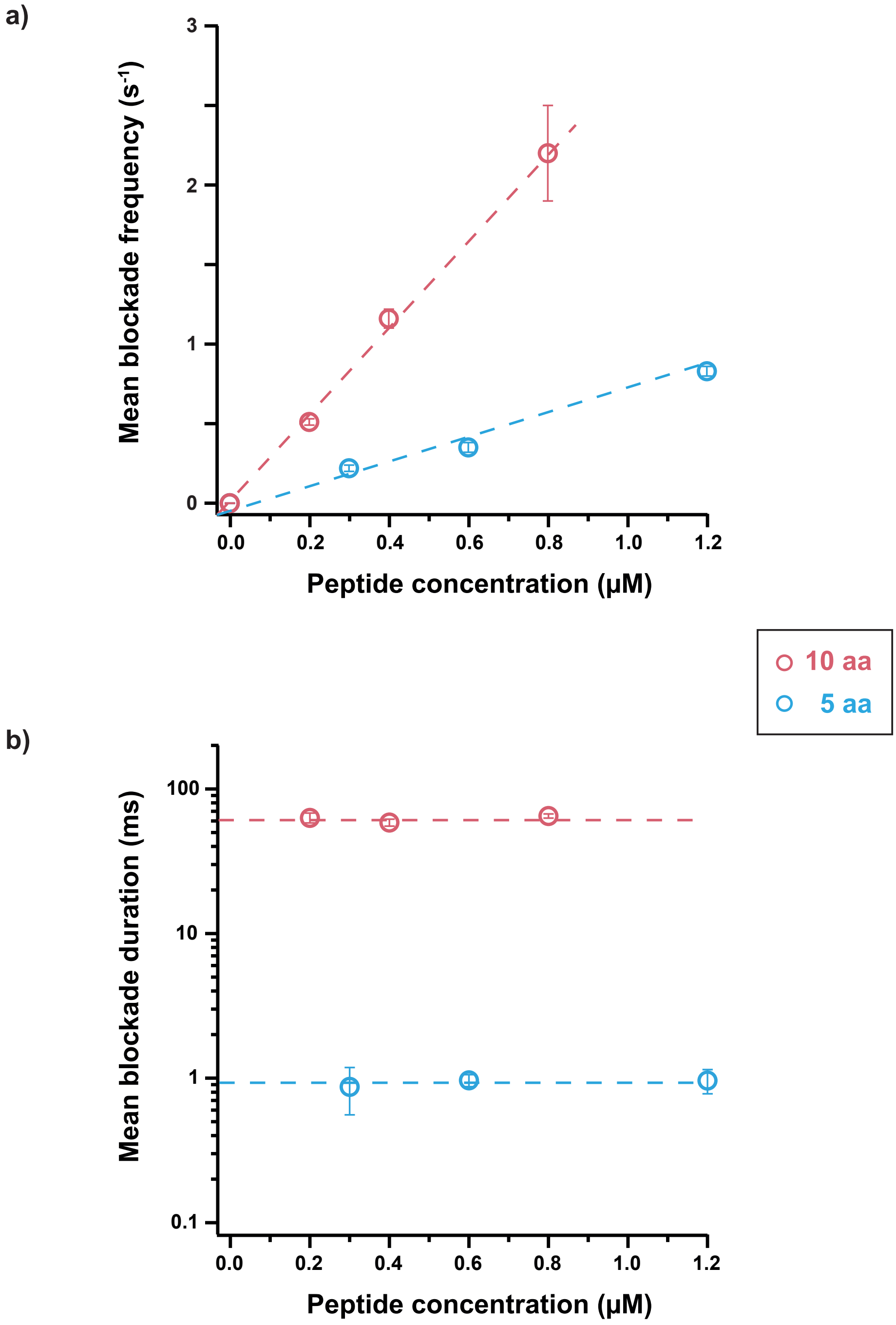

Supplementary Figure 6: **Effect of peptide concentration on the interaction of aerolysin nanopore with arginine peptides of different lengths.** (a) Mean blockade frequency *versus* peptide concentration in the case of a solution of 10 amino acids long peptides (red) and in the case of a solution of 5 amino acids long peptides (blue). Dashed lines are linear fits. The linear increase of the mean blockade frequency f with peptide concentration c is interpreted as a reversible peptide/aerolysin bimolecular reaction, with well-defined kinetic reaction constants. The slope of the linear fit gives the rate constante of association $k_{on}(M^{-1}s^{-1}) = \frac{f}{c}$ for each peptide length. (b) Mean blockade duration *versus* peptide concentration in the case of a solution of 10 amino acids long peptides (red) and in the case of a solution of 5 amino acids long peptides (blue). The mean blockade duration τ is independent of peptide concentration. The inverse of τ is defined as the rate constant of dissociation $k_{off}(s^{-1}) = 1/\tau$. The equilibrium association constant is defined as $K(M^{-1}) = k_{on}/k_{off}$, which gives the affinity between the peptides and the aerolysin nanopore. The corresponding standard free energy is defined as $\Delta G^0 = -k_BT\ ln(\frac{1}{K})$ where k_BT is the thermal energy $\approx 4\ 10^{-21}$J at 20°. The data were acquired in KCl 4M HEPES 5mM pH=7.5 at -50 mV and at 20°C.

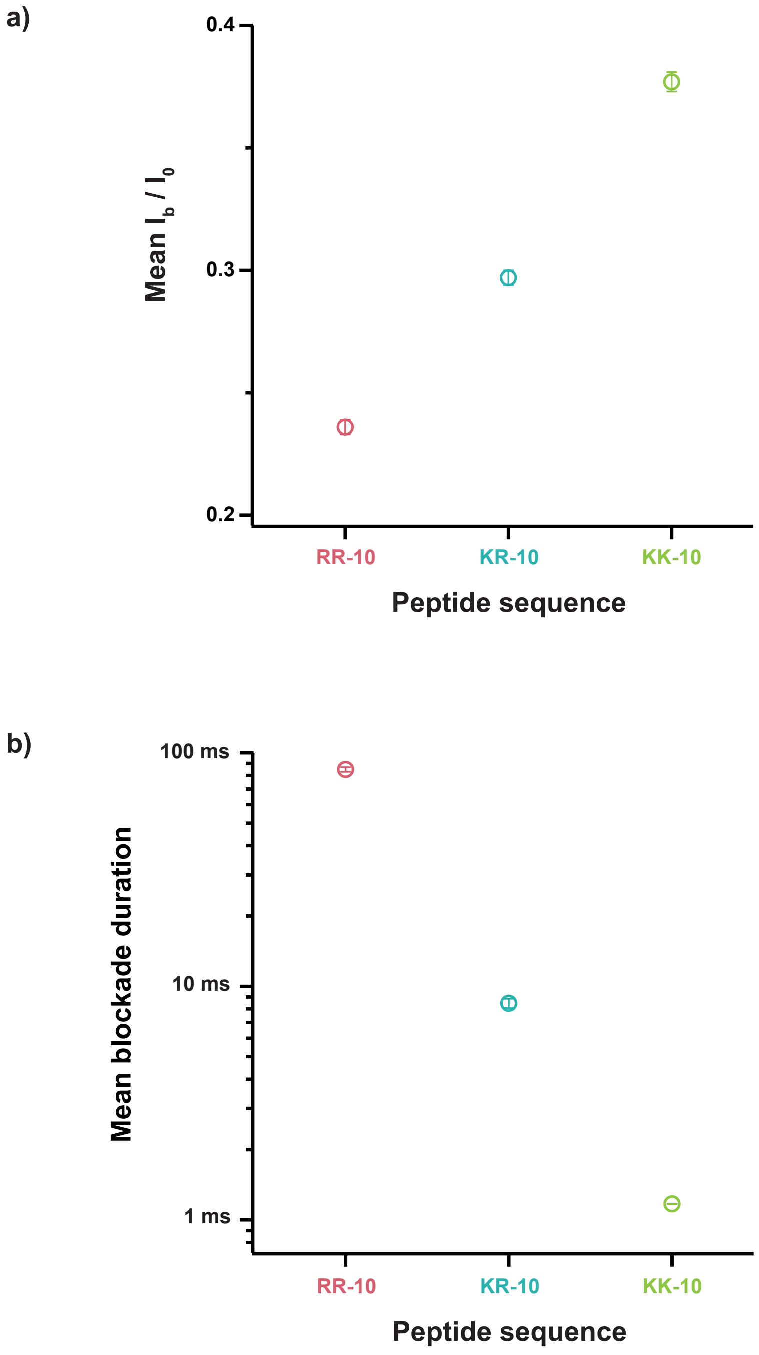

Supplementary Figure 7: **Discrimination of 10 amino acids long peptides of different sequences.** (a) Mean relative blockade current I_b/I_0 *versus* peptide sequence and (b) mean blockade duration *versus* peptide sequence in the case of the interaction of aerolysin nanopore with (from left to right) a solution of RR-10 peptides, with a solution of KR-10 peptides and with a solution KK-10 peptides. The data were acquired in KCl 4M HEPES 5mM pH=7.5 at -50 mV and at 20°C.

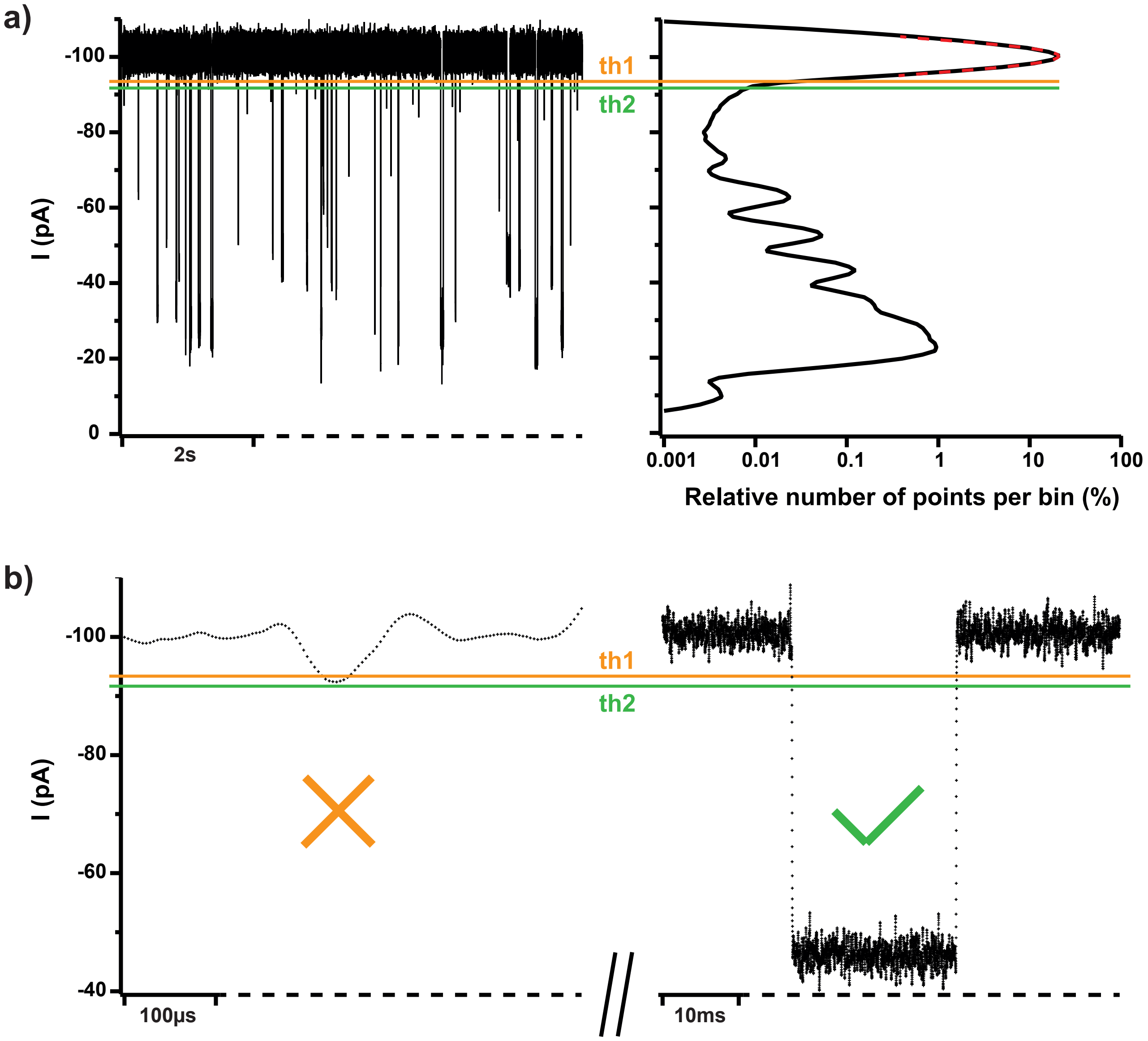

Supplementary Figure 8: **Analysis method for the detection of current blockades in a nanopore current recording.** (a, left) Portion of a typical current *versus* time recording through aerolysin nanopore in presence of an equimolar mixture (1μM) of arginine peptides of different lengths (5, 6, 7, 8, 9 and 10 amino acids) in KCl 4M HEPES 5mM pH=7.5 at -50 mV and at 20°C (negative current values under a negative applied voltage). (a, right) Histogram of the current values of the complete current *versus* time recording ($\approx$ 15 min) from which the recording portion in (a, left) was extracted. The largest peak of the histogram corresponds to the open-pore current. The mean open-pore current value I_0 and its standard deviation σ_0 are obtained by a gaussian fit of this peak (red dashed curve). The detection of current blockades is based on a two-thresholds method: a possible current blockade event starts when the nanopore current value becomes smaller than a first current threshold $th_1 = I_0 - 4\sigma_0$, and ends when the nanopore current returns to a value greater than th_1. If the mean current value during the event is greater than a second current threshold $th_2 = I_0 - 5\sigma_0$, the event is not considered as a peptide-induced current blockade and is rejected (as illustrated in (b, left)). In contrast, if the mean current value during the event is smaller than th_2, the event is considered as a peptide-induced current blockade (as illustrated in (b, right)).

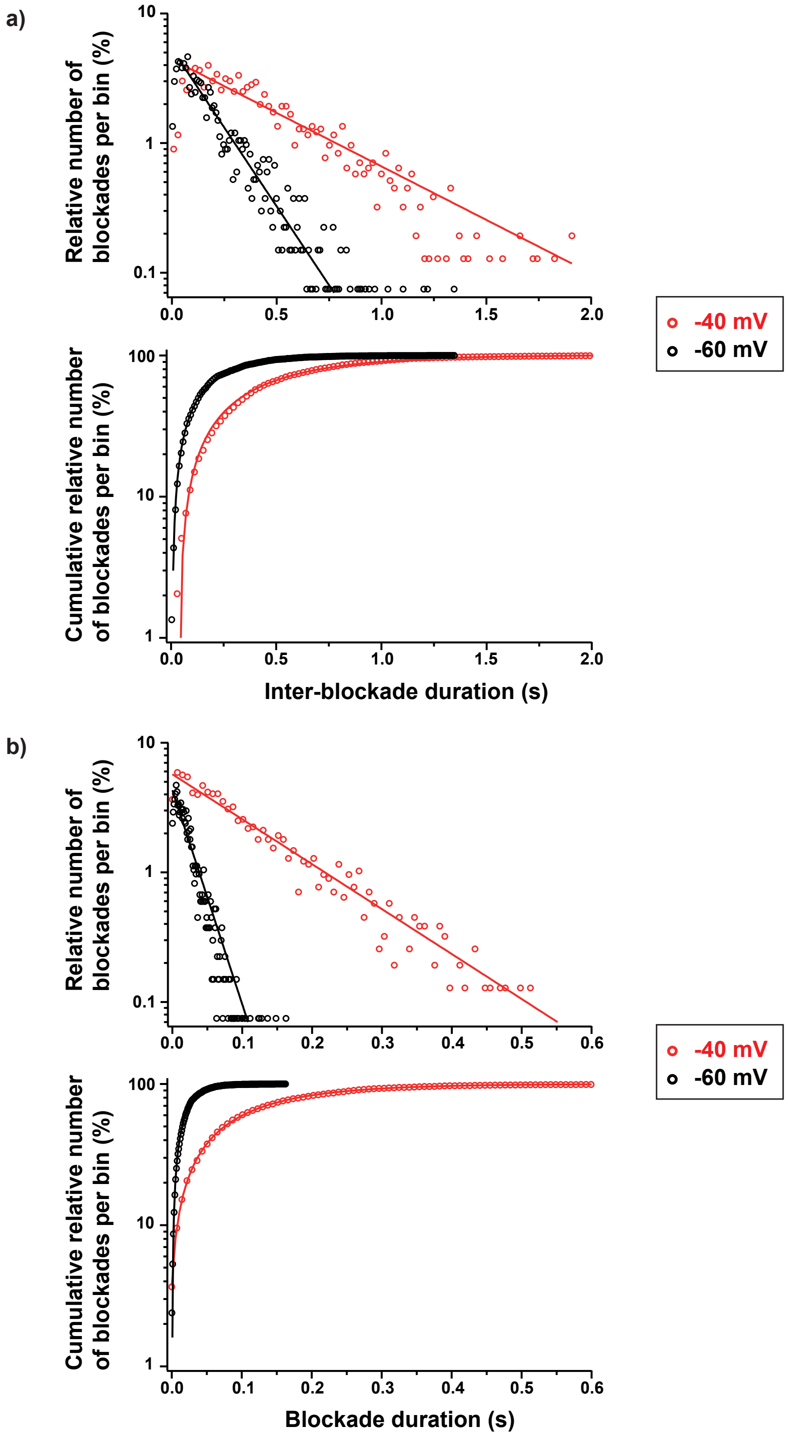

Supplementary Figure 9: **Statistical analysis of the inter-blockade duration and blockade duration distributions in the case of the interaction of aerolysin nanopore with a solution of 1.2 μM of 10 amino acids long arginine peptides.** (see the Methods section for details) (a, top) Histograms of the inter-blockade duration values at two different voltages: -40 mV (red) and -60 mV (black). (a, down) Cumulative histograms of the inter-blockade duration values at two different voltages: -40 mV (red) and -60 mV (black). Solid lines are single exponential fits from which the mean inter-blockade duration values are extracted: 526 +/- 24 ms (top) and 426 +/- 4 ms (down) at -40 mV ; 178 +/- 6 ms (top) and 181.9 +/- 0.7 ms (down) at -60 mV. (b, top) Histograms of the blockade duration values at two different voltages: -40 mV (red) and -60 mV (black). (b, down) Cumulative histograms of the blockade duration values at two different voltages: -40 mV (red) and -60 mV (black). Solid lines are single exponential fits from which the mean blockade duration values are extracted: 125 +/- 3 ms (top) and 114.9 +/- 0.2 ms (down) at -40 mV ; 26 +/- 1 ms (top) and 20.5 +/- 0.1 ms (down) at -60 mV. The data were acquired in KCl 4M HEPES 5mM pH=7.5 at 20°C.

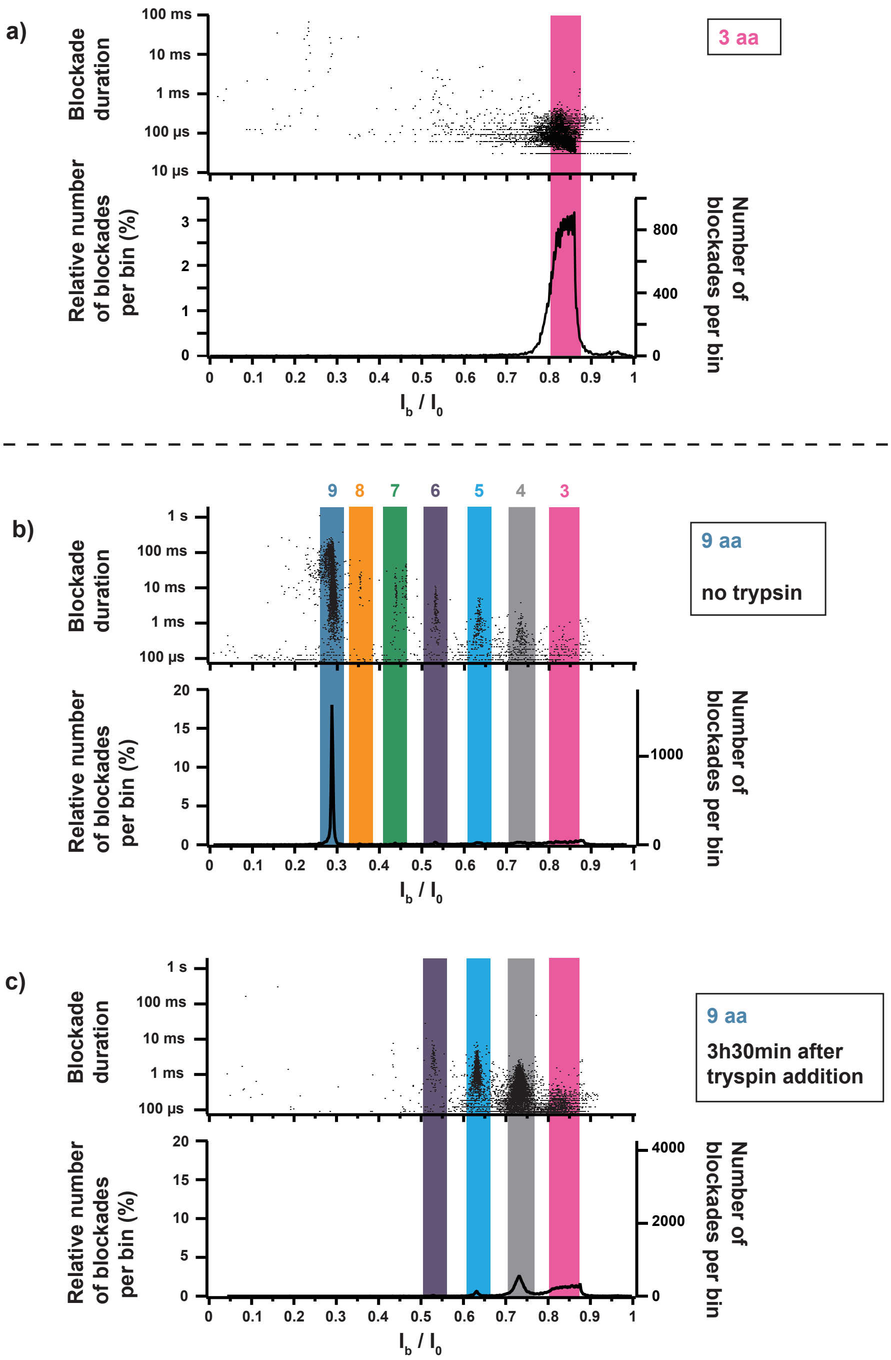

Supplementary Figure 10: **Detection and identification of 3 amino acids long arginine peptides.** Scatter plot of blockade duration versus relative blockade current I_b/I_0 (top of each subfigure) and histogram of the relative blockade current I_b/I_0 values (bottom of each subfigure) in the case of the interaction of aerolysin nanopore with a solution of 3 amino acids long arginine peptides (a) or in the case of the interaction of aerolysin nanopore with a solution of 9 amino acids long arginine peptides in absence of trypsin (b), and 3 hours and 30 minutes after trypsin addition (c). (a) The analysis of the 3 amino acids long arginine peptides reveals a population at a mean I_b/I_0 value of 0.83 +/- 0.02. (b) The analysis of the 9 amino acids long arginine peptides reveals that the overwhelming majority of blockades correspond to 9 amino acids long peptides. (c) After 3 hours and 30 minutes of trypsin activity, the 9, 8 and 7 amino acids long peptides populations have disappeared in favour of an increase of the proportion of blockades corresponding to shorter peptides indicating the trypsin cleavage of long peptides into shorter ones, more particularly into 6, 5 and 4 amino acids long arginine peptides. In (b) and (c), in addition to the emerging 6, 5 and 4 amino acids long peptides populations, another population is detected at a mean I_b/I_0 value of 0.83 +/- 0.02 (pink) allowing to identify this population as corresponding to 3 amino acids long arginine peptides. The data were acquired in KCl 4M HEPES 5mM pH=7.5 at 20°C.

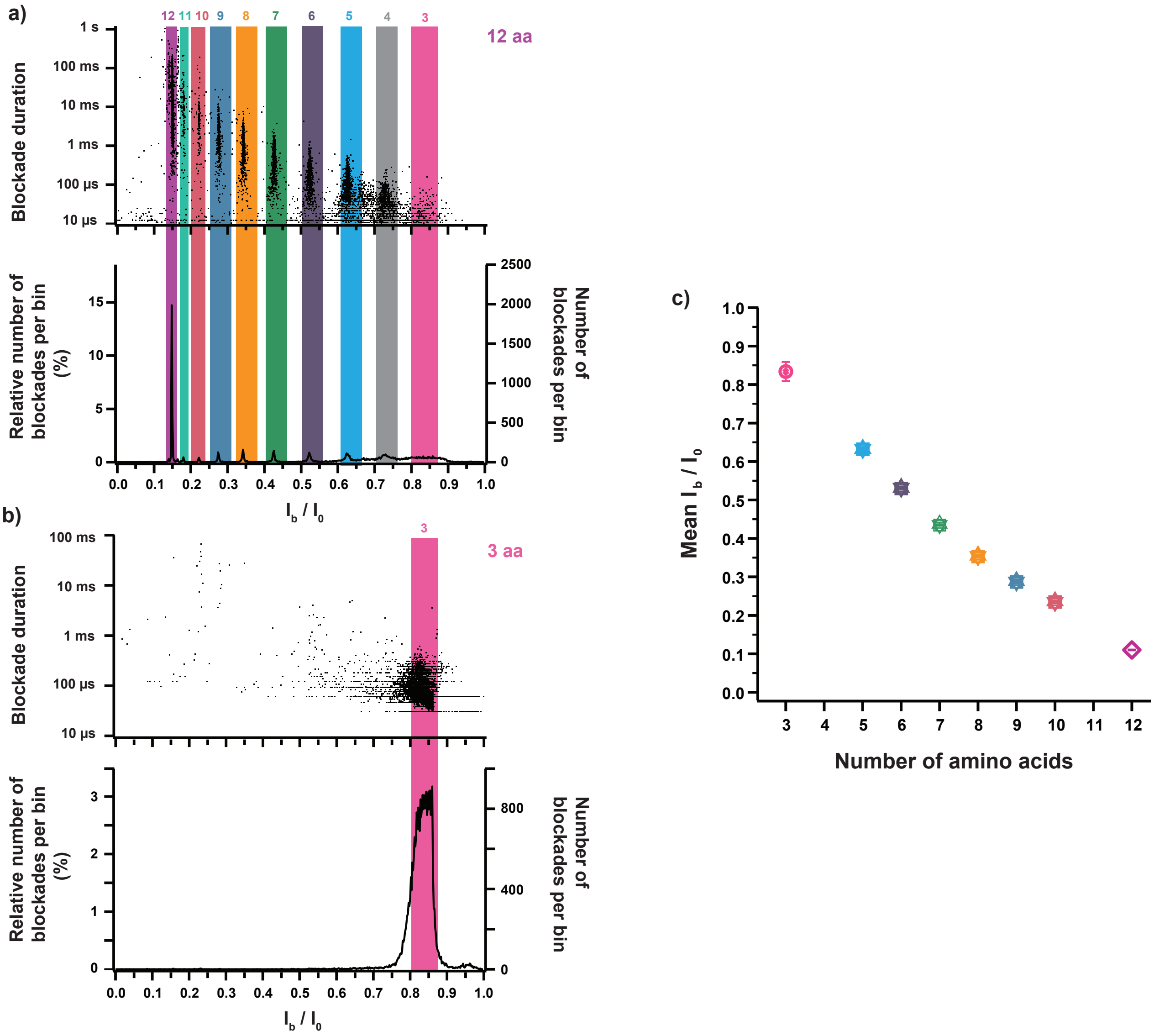

Supplementary Figure 11: **Detection and identification of 12 and 3 amino acids long arginine peptides.** (a, b) Scatter plot of blockade duration versus relative blockade current I_b/I_0 (top of each subfigure) and histogram of the relative blockade current I_b/I_0 values (bottom of each subfigure) in the case of the interaction of aerolysin nanopore with : (a) a solution of 12 amino acids long arginine peptides or (b) a solution of 3 amino acids long arginine peptides. Each histogram and scatter plot corresponding to a given peptide length exhibits a single main I_b/I_0 population, allowing to identify the arginine peptides of 12 and 3 amino acids. In addition to the 12 amino acids long arginine peptide population, 9 others populations are observed and attributed to 11, 10, 9, 8, 7, 6, 5, 4 and 3 amino acids long arginine peptides. (c) Mean relative blockade current I_b/I_0 as a function of the number of amino acids in the case of the interaction of aerolysin nanopore with an equimolar mixture of arginine peptides of different lenghts (5, 6, 7, 8, 9 and 10 amino acids) (see Supplementary Figure 1), and with 3 and 12 amino acids long arginine peptides analysed independently. The data were acquired in KCl 4M HEPES 5mM pH=7.5 at 20°C.
